# Nationwide survey of neonatal transportation practices in Italy

**DOI:** 10.1186/s13052-019-0640-z

**Published:** 2019-04-18

**Authors:** Maurizio Gente, Roberto Aufieri, Rocco Agostino, Tiziana Fedeli, Maria Grazia Calevo, Paolo Massirio, Carlo Bellini, on behalf of the Neonatal Transport Study Group of the Italian Society of Neonatology (SIN), S. Schettini, F. Raimondi, M. Carpentieri, M. Panico, S. Demarini, L. Cattarossi, F. Ferrari, G. Gargano, M. Gente, A. Dotta, C. Bellini, F. Mosca, S. Martinelli, E. P. Villani, L. Bernardo, P. Tagliabue, M. Stronati, M. Barbarini, R. Bellù, M. Agosti, G. Chirico, G. Mangili, C. Flumini, V. Santillo, F. Ferrero, D. Farina, M. Vivalda, E. Bertino, G. Guala, A. Marra, P. Gancia, D. Gazzolo, R. Magaldi, A. Gatta, M. Bisceglia, F. Tumminelli, I. Barbieri, A. Arco, G. Sulliotti, C. Cacace, M. Moroni, B. Tomassini, A. Boldrini, P. Biver, H. Messner, E. Baraldi, D. Trevisanuto, E. M. Padovani

**Affiliations:** 1 Department of Pediatrics and Infant Neuropsychiatry, Neonatal Emergency Transport Service, Sapienza University of Rome, Rome, Italy; 2 Division of Neonatology and Neonatal Intensive Care Unit, ASL Roma 2 – Ospedale Sant’Eugenio, Rome, Italy; 3 Division of Neonatology, Villa Margherita Private Nursing Home, Rome, Italy; 4 Neonatal Intensive Care Unit, Fondazione MBBM, Monza, Italy; 5 Department of Epidemiology, Biostatistics and Committees, IRCCS Istituto Giannina Gaslini, Genoa, Italy; 6 Neonatal Intensive Care Unit, Neonatal Emergency Transport Service, Department Mother&Child, IRCCS Istituto Giannina Gaslini, Largo G. Gaslini, 5, 16147 Genoa, Italy

**Keywords:** Infant, newborn, Intensive care, neonatal transport, Perinatal care, Regional medical programs, Health services accessibility

## Abstract

**Background:**

Despite regionalization of perinatal care provides for the “in utero” transfer of high-risk pregnancies, there will always be a number of neonates who undergo acute inter-facility transport. The presence of a well-organized Neonatal Emergency Transport Service (NETS) can prevent and reduce risks of transportation, especially for very preterm infants, and is therefore mandatory for any program of regionalization of perinatal care. Italian National Health System is highly decentralized and Regions are autonomous to structure, plan and delivery their regional health services. Consequently, organization models and resources available vary widely and significant regional differences in access and quality of health services have been reported in the past years. A national survey was conducted in 2015 by the neonatal transport study group of the Italian Society of Neonatology with the aim to describe neonatal transfer practices and to assess the Neonatal Emergency Transport Services (NETS) status in the 20 Italian regions.

**Methods:**

A questionnaire regarding neonatal transfer practices and NETS activity for the previous year (2014) was sent to the 44 NETS operating in the 20 Italian regions. Demographic data were obtained from the Italian National Statistical Institute (ISTAT).

**Results:**

The overall survey response rate was 100%. In 2014, only 12 (60%) of the 20 Italian regions were fully covered by NETS, 3 (15%) regions were partially covered, while neonatal transport was not available in 5 (25%) regions. Overall, in 2014, the 44 NETS operating in Italy transported a total of 6387 infants, including 522 (8.17%) having a gestational age < 28 weeks.

**Conclusions:**

The organization of NETS in Italy is devolved on a regional basis, resulting in a large heterogeneity of access and quality to services across the country. Where available, NETS are generally well-equipped and organized but limited volume of activities often cannot guarantee adequate levels of skills of personnel or an appropriate cost-efficiency ratio. The regions reported with lack of NETS have managed, or are trying, to fill the gap, but continuing efforts to reduce regional differences in the availability and quality of services are still needed.

## Background

In a network aimed at the regionalization of perinatal care, high-risk pregnancies should be transferred “in utero” in order to minimize risks to both the mother and the neonate [1, 2]. However, it is not always feasible to predict and prevent all the conditions possibly requiring neonatal care that cannot be provided in the referral center and there will always be a number of neonates who undergo acute inter-facility transport [3]. Neonatal transport represents an additional risk factor for a critically ill patient, especially for very preterm infants [1, 4]. Therefore, the presence of a well-organized Neonatal Emergency Transport Service (NETS) is mandatory for a perinatal regional network, as it represents the link between birth centers and neonatal intensive care units (NICUs) and can reduce risks of transportation, especially for very preterm infants [5, 6]. In the past years, a program of regionalization of perinatal care was implemented and NETS have been progressively activated in Italy [7, 8].

Regional governments in Italy have the autonomy to legislate issues regarding healthcare, thus resulting in regional variations in resources and models of organizations with differences in health care quality and outcomes, as measured by several indicators and reported by the Organisation for Economic Co-operation and Development (OECD) and the European Observatory on Health Systems and Policies [9, 10].

The aim of this study was to describe neonatal transfer practices and to assess the current organization of NETS in Italy. For this purpose, in 2015, a national survey was carried out by the neonatal transport study group of the Italian Society of Neonatology (SIN) under the auspices of the Italian Ministry of Health.

## Methods

### Survey

A survey regarding NETS activity was conducted in 2015, at all 44 existing Italian NETS. An exploratory, descriptive design, including a survey questionnaire, was adopted to maximize sample size and facilitate data collection. A multiple-choice questionnaire consisting of 20 questions regarding NETS organization and activity data for the previous year (2014) was designed by the SIN neonatal transport study group. The aim of the questionnaire was mainly to identify and describe NETS status, organization, coverage and activity; therefore, questions regarding outcomes of the transferred newborns were not included in this survey.

The questionnaire included questions about: a) annual volume of NETS activity, including the number of primary and back transports, number of transported newborns ≤28 week gestational age (GA), number of transports of infants over 28 days of life or 44 weeks of corrected GA for preterm infants, and average time of each transport; b) type of organization (i.e., number of unit-based teams, dedicated teams, and free-standing independent transport services); c) policies for quality evaluation, training and education d) vehicles used, including the availability of a helicopter for air transports.

The survey received approval from the SIN institutional review committee. The web-based survey tool Survey Monkey (http://www.surveymonkey.com/) was used and emails were sent to the person in charge of the NETS at each institution asking them to participate in the survey and to fill in the online questionnaire. Filling in the questionnaire implied consent to participate.

Filled in questionnaires were checked for invalid responses or missing data. Requests for missing information were made to the NETS directors by the secretary of the SIN Neonatal Transport study group by telephone. When all the questionnaires were ready the data were transferred to an electronic database and evaluated by the SIN neonatal transport study group.

Demographic data were obtained from governmental sources (ISTAT, Istituto Nazionale di Statistica - National Statistical Institute, available at http://www.istat.it/). Data about the number of birth centers and the number of birth centers with less than 500 births/year operating in 2014 were obtained, for each region, by the document of the Italian Ministry of Health: “Attuazione delle azioni previste dall’accordo del 16 dicembre 2010. Linee di indirizzo per la promozione e miglioramento della qualità, della sicurezza e dell’appropriatezza degli interventi assistenziali nel percorso nascita e per la riduzione del taglio cesareo. Monitoraggio al 31 dicembre 2014”, with the exception of Lazio and Campania, where data obtained from the available regional reports have been used [11–13].

### Italian administrative organization

Italy is subdivided into 20 regions, five of which have a special autonomous status. The Italian State has run a universal public national healthcare system since 1978, which is managed by the Ministry of Health and administered on a devolved regional basis. Each regional government is responsible for organizing its own healthcare system. The results were grouped by region to allow a comparison among regions and not among individual NETS.

### Statistical analysis

Statistical analysis consisted of descriptive statistics and the evaluation of categorical variables; continuous variables are presented as mean values ± standard deviation (SD), whereas they are presented as absolute and relative frequencies where needed. Data are provided both as absolute numbers and relative frequencies. The χ^2^ test or Fisher’s exact test were used to compare differences in categorical variables between groups. A *p*-value less than 0.05 was considered statistically significant, and all *p*-values were based on two-tailed tests. Statistical analysis was performed using SPSS for Windows (SPSS, Inc., Chicago, IL).

## Results

The response rate for the questionnaire was 100% (44/44). After a single request for missing data the 44 questionnaires were fully filled in, thus all of them were included in the analysis. The results, including demographic data for each region, are reported in Table 1.

**Table 1:** Summary of the nationwide survey results on Italian NETS. All data refers to the year 2014

Region	Overall Births	Inhabitants at 31st December 2014	Surface (km^2^)	30-day mortality rate ‰	NICUs	NICUs with > 90 VLBW (< 1500 g) admissions per year	NETS	Births per NICU	Births per NETS	NETS per km^2^	Maternity wards	Maternity wards with ≤ 500 births per year	Ratio Maternity wards per NETS	Total Neonatal transports	Primary Neonatal transports	Neonatal Back-transports	Neonatal transports of infants with GA ≤ 28 weeks	NTI (%)	NTI for Primary transports (%)	Average time of transports (minutes)	Type of Ambulance (Dedicated-not Dedicated)	NETS Helicopter availability
Piemonte	34,637	4,406,860	25,387	2.5	7	1	7	4948	4948	3626	29	7	4.14	363	314	49	8	1.04	0.90	125	5–2	No
Valle d’Aosta	1119	127,390	3260	2.6	0	–	0	–	–	–	1	0		–	–	–	–	–	–	–	–	–
Lombardia	86,239	10,004,794	23,863	2.7	17	2	11	5072	7839	2169	70	10	6.36	932	851	81	44	1.08	0.98	105	4–7	Yes
Trentino-Alto Adige	10,379	1,058,505	13,606	3.5	2	0	2	5189	5189	6803	13	7	6.50	173	131	42	1	1.66	1.26	167	0–2	Yes
Veneto	40,629	4,916,815	18,407	2.5	7	2	2	5804	20,314	9203	39	9	19.50	424	283	141	56	1.04	0.69	148	0–2	Yes
Friuli-Venezia Giulia	9177	1,060,000	8240	1.6	2	0	2	4588	4588	4120	11	2	5.50	157	121	36	2	1.71	1.31	90	2–0	Yes
Liguria	10,749	1,573,837	5416	2.6	1	1	1	10,749	10,749	5416	11	0	11	243	164	79	9	2.26	1.52	150	1–0	Yes
Emilia-Romagna	36,668	4,446,584	22,452	3.5	8	0	2	4583	18,334	11,226	29	8	14.50	36	26	10	2	0.09	0.07	80	0–2	No
Toscana	29,118	3,745,983	22,987	3.2	6	1	3	4853	9706	7662	25	5	8.33	363	357	6	170	1.24	1.22	102	1–2	Yes
Umbria	7015	891,848	8464	3.3	2	0	0	3507	–	–	11	7		–	–	–	–	–	–	0	–	–
Marche	12,363	1,544,237	9401	2.4	1	0	1	12,363	12,363	9401	14	1	14	212	76	136	5	1.71	0.61	190	0–1	Yes
Lazio	50,360	5,886,977	17,207	3.6	5	1	2	10,072	25,180	8603	44	12	22	1331	1316	15	134	2.64	2.61	120	2–0	Yes
Abruzzo	10,534	1,327,171	10,831	4.4	1	0	0	10,534	–	–	12	3		–	–	–	–	–	–	–	–	–
Molise	2213	312,484	4460	4.0	1	0	1	2213	2213	4460	3	2	3	26	23	3	0	1.17	1.03	60	0–1	No
Campania	51,243	5,852,729	13,670	4.4	10	0	3	5124	17,081	4556	67	20	22.33	1597	1583	14	58	3.11	3.08	118	3–0	No
Puglia	33,191	4,079,278	19,540	4.9	7	0	2	4741	16,595	9770	35	6	17.50	157	157	0	4	0.47	0.47	65	2–0	Yes
Basilicata	4123	574,251	10,073	4.5	1	0	1	4123	4123	10,073	6	2	6	16	14	2	0	0.38	0.01	120	1–0	Yes
Calabria	16,490	1,972,149	15,221	5.3	2	0	0	8245	–	–	15	1		–	–	–	–	–	–	–	–	–
Sicilia	44,876	5,077,487	25,832	4.5	6	0	4	7479	11,219	6458	56	17	14	357	336	21	29	0.79	0.74	95	2–2	Yes
Sardegna	11,473	1,658,649	24,100	3.4	3	0	0	3824	–	–	17	9		–	–	–	–	–	–	–	–	–
ITALY	502,596	60,518,028	302,426	3.4	89	8	44	5647	11,688	6903	508	128 (25.2%)	11.55	6387	5752	635	522 (8.17%)	1.28	1.14	123 ± 80	23–21	33–11

### Regional coverage

**Fig. 1:**
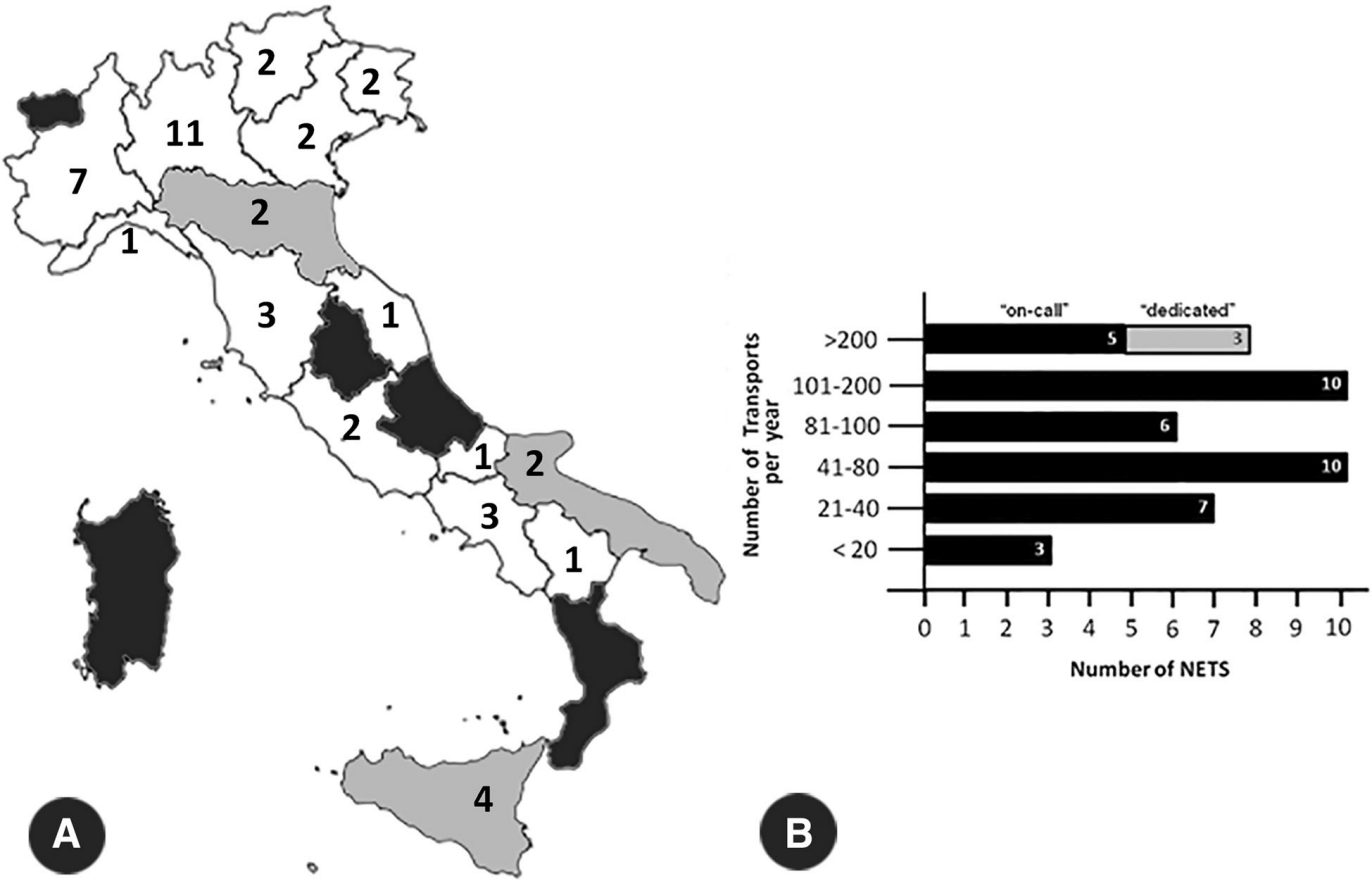
Geographical distribution and amount of activity of the 44 Italian NETS. Panel **a.** Geographical distribution of NETS in Italy, in 2014. Regional borders are shown. White regions were fully covered by NETS (Piemonte, Lombardia, Trentino-Alto Adige, Veneto, Friuli-Venezia Giulia, Liguria, Toscana, Marche, Lazio, Molise, Campania, and Basilicata); grey regions were partially covered (Emilia-Romagna, Puglia e Sicilia); NETS was not available in black regions (Valle d’Aosta, Umbria, Abruzzo, Calabria e Sardegna). Panel **b.** Number of transports per year of the 44 NETS, in 2014. All the three NETS organized on a dedicated model carried out more than 200 transports per year

Results showed that in 2014, among the 20 Italian regions only 12 were fully covered by NETS (Piemonte, Lombardia, Trentino-Alto Adige, Veneto, Friuli-Venezia Giulia, Liguria, Toscana, Marche, Lazio, Molise, Campania, and Basilicata), 3 regions (Emilia-Romagna, Puglia e Sicilia) were partially covered, while neonatal transport was not available in 5 regions (Valle d’Aosta, Umbria, Abruzzo, Calabria e Sardegna) (Fig. 1, panel a).

### NETS organization

The results of the survey showed how all 44 NETS guaranteed 24/7 service coverage. 41/44 NETS were organized as an on-call service, while three were fully dedicated services. Each of the forty-one on-call NETS was linked to a NICU which provided a senior neonatologist and a neonatal nurse for each transport.

### NETS activity

Overall, in 2014, the 44 NETS in Italy transported a total of 6387 infants, 522 (8.17%) of whom were of GA < 28 weeks, and 635 (9.94%) who were back-transported infants. Median regional transport time was 123 min (minimum-maximum range 60–190).

**Fig. 2:**
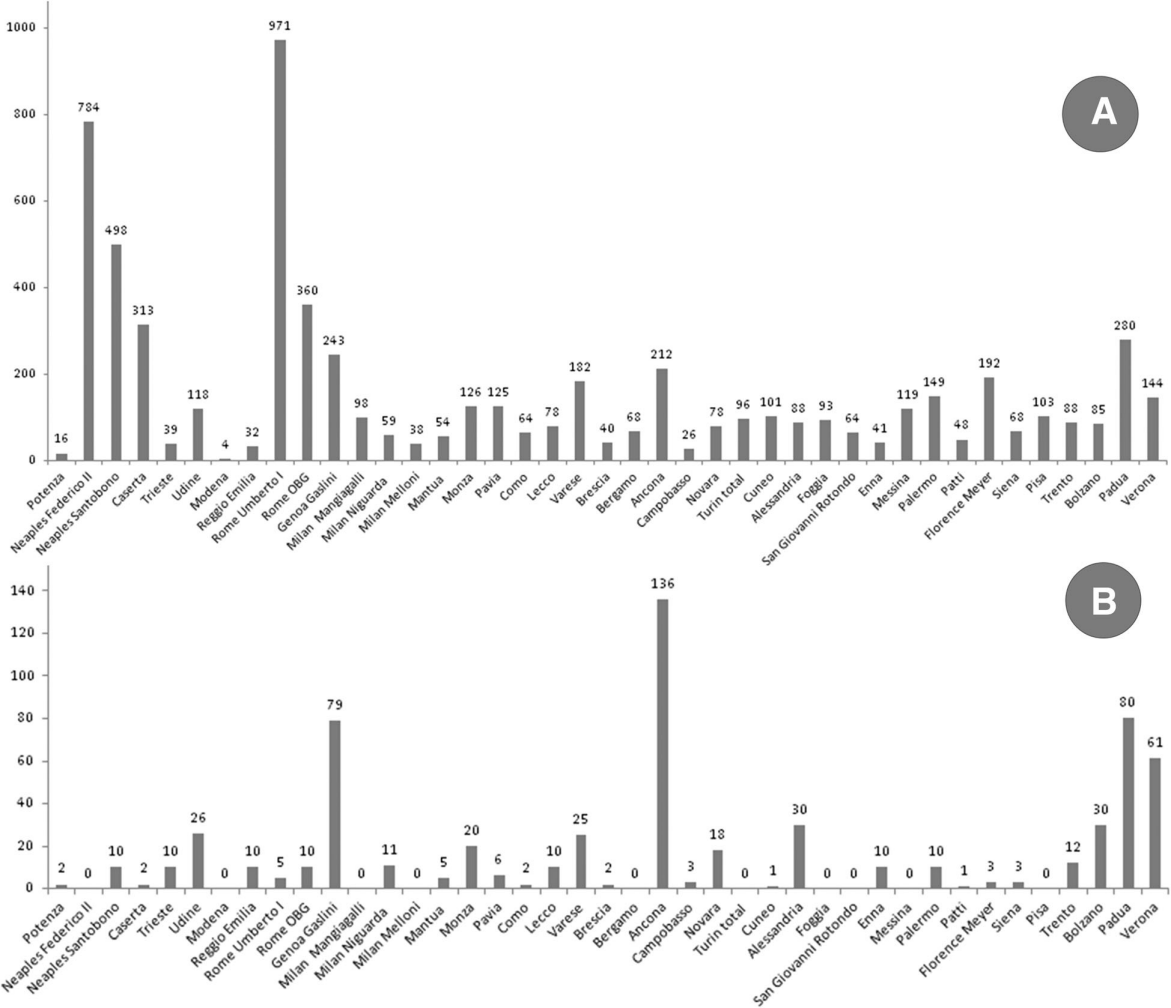
Distribution of transports in Italy. Total number of transports within various Italian NETS in 2014; note that the four Turin NETS were grouped. Panel **a**: total number of transports. Panel **b**: total number back-transports

Among the 41 on-call NETS, 3 carried out fewer than 20 transports per year, 7 carried out between 21 and 40, 10 between 41 and 80, 6 between 81 and 100, a further 10 between 101 and 200 and only 5 on-call NETS carried out more than 200 transports per year. All the 3 dedicated services carried out more than 200 transports per year (Fig. 1, panel b). The number of total transports and back-transports varied widely within the Italian territory (Fig. 2).

21/ 44 NETS also provided transportation for infants and children older than 28 days of life or 44 weeks of corrected GA for preterm. These patients were transferred both from the same hospitals where the services were based (9% from the NICUs, 23% from other units) and from other hospitals (68% of cases). T Most of them weighed less than 6 kg (67%) and in 48% of cases were infants below 3 months of age. However, 14% of these transports involved children weighing more than 10 kg and who were over 1 year of age.

The neonatal transport index (NTI) i.e., the number of neonates transferred per 100 live births (back-transports included or excluded), is reported in Table 1. We performed a broad statistical analysis and found that the comparison between the maternity wards per NETS ratio vs NTI for primary transports and the comparison between the rate of hospitals with ≤500 births per year vs 30-day mortality was significant (*p*-value < 0.00001 and 0.00002, respectively) while the comparison between the number of neonatal transports vs 30-day mortality was slightly significant (p-value 0.007). No other statistically significant results were observed.

### Vehicles and air transport

Dedicated ambulances for neonatal transport were owned by 23/44 NETS (52.2%). Fifteen of the 21 NETS based at a level III NICU, where a dedicated ambulance for neonatal transport was not available, reported that they were unable to purchase one owing to financial constraints.

Air transport by helicopter was not available for all NETS, as it was only carried out in 10 of the 15 regions covered by NETS. Fixed wing air transport was not available on a routine basis all over Italy. However, the Italian Air Force allowed NETS to use their aircraft for urgent cases, mainly for transfers of neonates with congenital heart defects or surgical emergencies.. This occurred especially for transports from the islands of Sicilia and Sardegna towards cities with hospitals providing pediatric/neonatal surgery, cardiothoracic surgery or other special care.

### Quality evaluation, training and education

A dedicated NETS database was available in 42/44 services; specific guidelines were edited by 43/44 NETS and in one case by the “112 Emergency Service” (118 at the time of the Survey), (i.e., 911 in the USA and 999 in the UK). Regular auditing was performed by 36/44 NETS; in two services through an agreement with the “112 Emergency Services”. No auditing was performed on a regular basis by the remaining 8 NETS. Training and education activities were provided at 40/44 NETS, at 2 NICUs linked to NETS, and at one 112 Emergency Service.

## Discussion

Organized NETS activity in Italy became available during the eighties [14], but it was during the nineties that NETS coverage mainly spread throughout Italy, with 10 of the 20 Italian regions reaching regional coverage > 50% in 1999 [9]. The agreement of the State-Regions Conference in December 2010 (also known as “birth path” or “percorso nascita”) provided guidance to standardize pregnancy and childbirth practices at regional level, including the adoption of perinatal networks based on the “hub and spoke” organizational model that guarantee the presence of Maternal and Neonatal Emergency Transport Services [15]. However, our study shows how in 2014 neonatal transport was still not available in an organized form in five Italian regions, though a NETS is not strictly required in Valle d’Aosta since there is only one perinatal center in that region.

The NTI is considered an indicator of the quality of regionalization of perinatal care [16]. In countries where perinatal care is highly regionalized, like in the UK, the NTI can be as low as 1% [17, 18]. Whereas in the past, in areas with heterogeneous distribution of obstetrics units, the NTI reached values of about 10%, e.g., in the Loire-Atlantique region in France [18] or in Portugal [19]. Our survey showed that regions which were fully covered by NETS, in 2014, had a mean NTI for primary transports (back-transports excluded) of 1.27 ± 0.84 and that the three other regions with partial NETS coverage had a mean NTI for primary transports of 0.43 ± 0.34. The two regions with the highest NTI for primary transports were Campania (NTI 3.08) and Lazio (NTI 2.61), both regions with full NETS coverage. This result can be explained both by the high number of birth centers that are currently active in these regions (67 in Campania and 44 in Lazio) and by the number of maternity wards carrying out ≤500 births per year (20 in Campania and 12 in Lazio, i.e., 29.9 and 27.3%, of birth centers, respectively). In 2014, in Italy, 25.2% of birth centers had an activity of less than 500 births per year, this data increases to 35.7% if we consider only the five regions without NETS coverage (Table 1).

The results of this survey also showed that most NETS (59.1%) carried out fewer than 100 transports per year (Fig. 1, panel b), which is a relatively low degree of activity when trying to provide its personnel with an adequate level of skills and experience and good cost performance [20].

Moreover, half of the level III perinatal centers did not have a specially equipped ambulance for neonatal transport (23/21, dedicated/non-dedicated), thus leading to difficulties in providing adequate transport of these vulnerable patients. Non-dedicated ambulances are usually not equipped with dedicated infant incubators, therefore, neonatal transport is usually carried out by simply placing an infant incubator on a stretcher designed for transporting adults [21–23]. It is well known that a lack of adequate equipment and low skill levels of the personnel involved in the transport increases the risk of serious adverse events in the transport of severely ill newborns [6]. Dedicated mechanical neonatal respirators, resuscitation equipments, standard vital-sign monitors, as well asdedicated twin-newborn [24, 25] and iNO [26] devices and are nowadays available and can improve safety and quality of care during transport.

The use of protocols, quality evaluation procedures and training and education programmes became widely adopted among NETS, with the exception of regular audits, which were not performed in 20% of NETS.

This survey demonstrated that there is no agreement regarding the limits to be applied to age and weight for neonatal transport, i.e., if the transport must be limited to the first 30 days of life, or if a broader approach is possible. We report that as much as 10 kg of weight or 1 year of age was the upper limits of transported patients, thus changing neonatal transport into pediatric transport.

This study has however some limitations that have to be pointed out. Collection and analysis of survey data have been laborious and required a considerable amount of time. NETS status is very recently changed in some Italian Regions. Abruzzo, Umbria and Calabria have activated their on-call NETS. Meanwhile for Sardegna NETS activations is foreseen soon, having already obtained deliberations from their regional governments [8]. The reported missing data together with the recent changes of NETS presence in some regions could represent several of the possible avenues for future researches.

## Conclusion

The inter-facility transport of severely ill and premature newborns is commonly performed in Italy by organized, generally well-equipped and well-trained NETS. The implementation of NETS, together with programs of regionalization of perinatal care have likely played a major role in reducing the neonatal mortality rate that has been observed in Italy in the last two decades (from 5.2‰ in 1998 to 3.4‰ in 2014). However, NETS coverage is still lacking in many densely populated areas and, where it is present, most of the NETS seem to have a volume of activity that is not sufficient to provide the personnel with an adequate level of skills and an appropriate cost-efficiency ratio. Moreover, many NETS report that, mainly due to financial constraints, there is still a lack of vehicles which are fully dedicated to neonatal transport, that would be of help to improve safety and quality of services.

Differences found in neonatal mortality rates, NTI, NETS availability, and NETS costs among regions seems to reflect the presence of different regional perinatal systems of organizations related to the autonomy of regional governments to legislate in matter of healthcare. It is envisaged that the full application of the State-Regions Conference agreement, along with the implementation of minimal standards of care and education programs driven by the national scientific societies, would lead toward the harmonization of perinatal care in Italy. These measures, however, constitute only a part of the continuing efforts required to improve outcomes and reduce the regional differences in the availability and quality of perinatal services. Periodical national and local audits are needed to evaluate and drive these quality improvement processes.

## Abbreviations

GA: Gestational Age
ISTAT: Italian National Statistical Institute
NETS: Neonatal Emergency Transport Services
NICU: Neonatal Intensive Care Unit
NTI: Neonatal Transport Index
OECD: Organisation for Economic Co-operation and Development
SD: Standard deviation
SIN: Italian Society of Neonatology
VLBW: Very low birth weight
